# Associations between postpartum depression and assistance with household tasks and childcare during the COVID-19 pandemic: evidence from American mothers

**DOI:** 10.1186/s12884-021-04300-8

**Published:** 2021-12-13

**Authors:** Theresa E. Gildner, Glorieuse Uwizeye, Rebecca L. Milner, Grace C. Alston, Zaneta M. Thayer

**Affiliations:** 1 Department of Anthropology, Washington University in St. Louis, St. Louis, MO USA; 2 Department of Anthropology, Dartmouth College, Hanover, NH USA; 3 Society of Fellows, Dartmouth College, Hanover, NH USA; 4 Ecology, Evolution, Environment & Society Program, Dartmouth College, Hanover, NH USA

**Keywords:** Mental health, Perinatal depression, Maternal wellbeing, Coronavirus, Social support, Childcare

## Abstract

**Background:**

The early postpartum period is recognized cross-culturally as being important for recovery, with new parents receiving increased levels of community support. However, COVID-19-related lockdown measures may have disrupted these support systems, with possible implications for mental health. Here, we use a cross-sectional analysis among individuals who gave birth at different stages of the pandemic to test (i) if instrumental support access in the form of help with household tasks, newborn care, and care for older children has varied temporally across the pandemic, and (ii) whether access to these forms of instrumental support is associated with lower postpartum depression scores.

**Methods:**

This study used data from the COVID-19 And Reproductive Effects (CARE) study, an online survey of pregnant persons in the United States. Participants completed postnatal surveys between April 30 – November 18, 2020 (*n* = 971). Logistic regression analysis tested whether birth timing during the pandemic was associated with odds of reported sustained instrumental support. Linear regression analyses assessed whether instrumental support was associated with lower depression scores as measured via the Edinburgh Postnatal Depression survey.

**Results:**

Participants who gave birth later in the pandemic were more likely to report that the pandemic had not affected the help they received with household work and newborn care (*p* < 0.001), while access to childcare for older children appeared to vary non-linearly throughout the pandemic. Additionally, respondents who reported that the pandemic had not impacted their childcare access or help received around the house displayed significantly lower depression scores compared to participants who reported pandemic-related disruptions to these support types (*p* < 0.05).

**Conclusions:**

The maintenance of postpartum instrumental support during the pandemic appears to be associated with better maternal mental health. Healthcare providers should therefore consider disrupted support systems as a risk factor for postpartum depression and ask patients how the pandemic has affected support access. Policymakers seeking to improve parental wellbeing should design strategies that reduce disease transmission, while facilitating safe interactions within immediate social networks (e.g., through investment in COVID-19 testing and contact tracing). Cumulatively, postpartum instrumental support represents a potential tool to protect against depression, both during and after the COVID-19 pandemic.

## Background

The early postpartum period represents a time of recovery and adjustment. In many cultures, the first 30-45 days are characterized by a set of common rituals, including rest periods and prescribed dietary and hygiene regimes believed to support maternal physical and mental health [1, 2]. Within the United States, however, less attention has been paid to postpartum health beyond the first few days following delivery [1]. Moreover, fewer formal mechanisms designed to support postpartum wellbeing, including federally mandated paid parental leave, exist within the U.S., potentially undermining maternal recuperation by exacerbating physical fatigue and compromising mental health ( [1, 2]). Postpartum health and mental wellbeing (including depression risk) are influenced by many biocultural factors, including experienced racism and racial disparities in resource access, pregnancy intendedness (i.e., whether the pregnancy was planned), previous experiences of trauma or poor physical and mental health, work-related anxiety, financial stress, and physical activity patterns [3–5]. Social support from partners, family members, and community members represents another important factor impacting postpartum recovery and health [3, 6–9], and some level of postpartum support is common in many American communities*.* New parents commonly receive support from family, friends, and neighbors as they adjust to life with a newborn. This support comes in different forms, including emotional and informational support [10–13]. However, another important type of postpartum assistance is instrumental support, which includes activities such as help with childcare and other household tasks (e.g., cleaning and housework preparation) [10–13].

Extended family members are generally an important source of instrumental support, often assisting with newborn care and daily household tasks during the postpartum period, allowing mothers to have needed time to themselves [14–16]. Instrumental support is therefore essential for supporting maternal wellbeing following childbirth, with evidence demonstrating that women consider this type of support from friends and family a critical part of their physical and emotional recovery [14, 15]. Conversely, mothers who receive less postpartum instrumental support exhibit an elevated risk for postpartum depression (PPD); for example, individuals in one study who reported receiving low levels of instrumental support were approximately five times more likely to develop PPD as mothers who reported high levels of instrumental support [17].

The positive impact of instrumental support on maternal wellbeing is not trivial, as new mothers may be at an especially high risk of developing depressive symptoms. The perinatal period is associated with a range of physical and physiological changes, often leading to increased levels of stress, fear, and anxiety with the transition to parenthood [8]. Postpartum individuals consequently exhibit elevated depression rates compared to the general public, such that as many as 1 in 8 women in the U.S. have been estimated to experience PPD symptoms ( [18, 19]). Postpartum depression can have serious effects not only on maternal quality of life (e.g., resulting in poor sleep quality, loss of appetite, lasting sadness, anxiety, thoughts and/or attempts to harm oneself or the baby), but may also negatively affect the infant. Maternal PPD can lead to difficulty in breastfeeding, poor maternal and infant bonding, and delays in multiple aspects of infant development which may increase the risk of early non-communicable disease onset during adulthood [20]. Thus, reducing PPD risk is important for both maternal and infant wellbeing, and enhanced support during the postpartum period may represent one important non-pharmaceutical strategy for supporting maternal mental health. Instrumental support in particular may protect against PPD, as this form of support has been shown to reduce the care burdens placed on mothers while also signaling that they are loved and valued ( [16, 21]).

Yet, the COVID-19 pandemic has disrupted daily life with implications for access to common forms of instrumental support postpartum. Travel restrictions tied with stay-at-home orders may prevent family and friends from visiting for the birth and early postpartum period [22], potentially decreasing the amount of help received with household tasks and newborn care. In addition, other sources of support may be disrupted, including access to school and daycare for older children [23, 24]. These school and childcare closures appear to especially impact mothers, who have disproportionately provided childcare and supervised remote learning during the COVID-19 pandemic [23, 24]. Cumulatively, these instrumental support disruptions may increase PPD risk among mothers living in the U.S., although this remains to be directly tested. Still, evidence in other countries suggests that PPD rates have risen during the pandemic, and that disrupted instrumental support may partly account for this pattern. For example, studies conducted in China and Italy indicate a high prevalence of PPD among individuals during the COVID-19 pandemic (30 and 44%, respectively), while reduced contact with loved ones was significantly associated with PPD [25, 26]. Further work is needed, however, to determine whether specific aspects of postpartum instrumental support may protect against PPD during the ongoing pandemic.

The impact of the COVID-19 pandemic on instrumental support access has likely varied over time, as rates of disease transmission and associated social distancing and travel recommendations fluctuate. Evidence indicates that stay-at-home orders imposed by local governments have changed over time, with widespread lockdowns at the start of the pandemic and a gradual easing of restrictions in subsequent months [27, 28]. Reduced mobility in the U.S. is clearly correlated with shelter-in-place restrictions, such that areas with official orders (more common in April than during the June-August) exhibited substantially greater reductions in movement compared to locations without stay-at-home orders [28, 29]. It therefore seems likely that pandemic-related changes to postpartum instrumental support have not remained constant, although this has not yet been fully explored. To address these issues, we use data from the COVID-19 and Reproductive Effects (CARE) study – an online survey of pregnant and postpartum persons living in the U.S. which assesses how the COVID-19 pandemic has affected maternal wellbeing. The aims of this study were to evaluate:

1. Whether the level of instrumental support (in the form of assistance with housework and newborn care or childcare for older children) reported within the first few weeks of the postpartum period has changed across the pandemic (April – November 2020).
2. Whether reported instrumental support during the COVID-19 pandemic is associated with lower maternal depression scores, as measured by the Edinburgh postnatal depression survey.

## Methods

### Study design

The COVID-19 And Reproductive Effects (CARE) study was posted on social media platforms (Facebook, Twitter) and distributed via email to contacts working in maternity care and public health. Pregnant persons over the age of 18 and living in the United States were invited to participate in a short survey assessing how the COVID-19 pandemic had impacted their healthcare and wellbeing. Participants who agreed to be re-contacted received a postnatal survey four weeks after their due date. The postnatal data presented here were collected between April 30 – November 18, 2020. This study received ethical approval from Dartmouth College (STUDY00032045) and all research was performed in accordance with the Declaration of Helsinki. Informed consent was obtained from all participants. The survey was administered using REDCap (Research Electronic Data Capture) hosted through Dartmouth College. REDCap is a secure web platform that facilitates the creation and management of online surveys for research studies [30, 31]. The survey completion rate (i.e., the percentage of those who consented to take the survey and actually went through to the end of the questionnaire) was 92.8% (1033/1113 participants). During the study period, there were 976 surveys collected that included responses for all study variables. Data on depression symptomatology and support systems were collected, along with other covariates known to influence depression risk.

#### Depression scores

Depression symptoms were screened using the gold-standard Edinburgh Postnatal Depression Survey (EPDS). The EPDS is a self-report 10-question instrument based on individual experiences in the previous seven days. This well validated scale is designed to measure various aspects of clinical depression, including reports of feeling guilty, disrupted sleep, low energy, inability to feel pleasure, and suicidal ideation. The responses are scored and summed, resulting in a participant score ranging from 0 (minimum, little indication of depressive symptoms) to 30 (maximum, high likelihood of depression) [32].

#### Postpartum social support

Participants were asked whether the COVID-19 pandemic had led to them receiving less help and support with household tasks and newborn care (yes/no).

#### Childcare support

Respondents were asked, “If you have other children, has/did the COVID-19 pandemic affect your access to childcare?” These data were analyzed for the subset of participants in the dataset with other children (*n* = 398) to determine whether childcare disruptions varied over the course pandemic or were related to maternal PPD. Specifically, individuals who responded that their other child(ren)‘s daycare had closed (either temporarily or permanently) or that their other child(ren) could no longer be cared for by others (e.g., a nanny or a relative) were coded as experiencing disrupted childcare. Conversely, participants who indicated that their other child(ren) were never cared for by others outside of their household or that their other child(ren) continued to go to daycare were coded as experiencing sustained childcare.

#### Birth date

Participants reported when they gave birth. This date was then used to calculate how far into the pandemic the participant gave birth. Specifically, the number of days between March 11, 2020 (the day the WHO officially declared COVID-19 a pandemic) and the day of birth was calculated, such that larger values reflect giving birth later in the course of the pandemic.

#### Postpartum duration

Time into the postpartum period may influence access to support systems (e.g., individuals may experience increased levels of support immediately after giving birth as compared to three months later). Additionally, postpartum period length also appears to influence the risk of developing depression [33, 34]. It is therefore important to account for postpartum duration when considering access to instrumental support and depression symptoms during the postnatal period. The number of days that had passed between giving birth and completing the postpartum survey was consequently calculated for each participant.

#### Maternal age

Past research indicates that maternal age is inversely related to depressive symptoms [35]. Thus, participants self-reported their age in years.

#### Race/ethnicity

Race/ethnicity has been linked with maternal depression risk, with minority populations exhibiting higher depression rates [35]. Participant race/ethnicity was therefore self-reported and measured according to the Office of Management and Budget Standards [36]. Native Hawaiian/Pacific Islander participants were re-classified as “Other” due to a small sample size (*n* = 3). This categorical variable was dummy coded during analysis, with “white” serving as the reference group.

#### Household income

Previous work indicates that higher income levels may protect against maternal depression [35]. Participants were thus asked to select their household income from the following options: Less than $10,000 (1); $10,000 – $19,999 (2); $20,000 – $34,999 (3); $35,000 – $49,999 (4); $50,000 – $74,999 (5); $75,000 – $99,999 (6); $100,000+ (7). A composite household income variable was created for analysis: < $49,999, $50,000 – $99,999, and $100,000+ (< $49,999 serves as the reference group in analysis).

#### Education

Lower education levels have been linked with increased depression risk during pregnancy [35]. Participants consequently selected their highest completed education from the following options: Some high school, no diploma (1); High school graduate, diploma or the equivalent (for example: GED) (2); Some college credit, no degree (3); Trade/technical/vocational training (4); Associate degree (5); Bachelor’s degree (6); Master’s degree (7); Professional degree (8); Doctorate degree (9). A composite education variable was created for analysis: less than a bachelor’s degree, a bachelor’s degree, or a degree beyond a bachelor’s degree (less than a bachelor’s degree serves as the reference group in analysis).

## Statistical analysis

Data analyses were conducted using Stata 14. All continuous variables exhibited normal distributions, with skewness values within + 1. Multicollinearity was not detected between any variables; all VIF values were in an acceptable range of 1.02-1.44. A plot of the linear regression model residuals versus fitted values did not indicate that heteroscedasticity was a concern. Five outliers were identified. Specifically, five participants exhibited extreme duration values between giving birth and completing the survey. Two respondents apparently completed the postpartum survey 13 and 2 days *before* giving birth, while another three waited months to complete the survey (i.e., 113, 114 and 129 days passed between giving birth and completing the survey). These five participants were consequently excluded from the analyses so that the analyses only accounted for depression scores and social support experiences within the first few months of giving birth (range 2-89 days following delivery). This resulted in a final sample size of 971 participants; a power analysis (for a linear multiple regression model with an estimated 6 predictors, power 0.80, and alpha 0.04) indicated that this sample size would have the sensitivity to detect an effect size f^2^ of 0.006, a very small effect. Study descriptive statistics were calculated, and regression analyses were conducted to test the study hypotheses. Results were considered statistically significant at *p* < 0.05.

Logistic regression analyses assessed whether later birth date (signifying later in the course of the pandemic) was significantly associated with an increased likelihood of reporting help with household work/newborn care due to the easing of initial lockdown measures. In other words, it was hypothesized that individuals giving birth later during the pandemic may have faced less severe shelter-in-place restrictions, and consequently would have been more likely to report that the pandemic had not reduced help received around the house. Conversely, the question about care for older children asked about care disruptions at any point during the pandemic. It was therefore hypothesized that later birth date would be associated with higher odds of reporting childcare disruptions (i.e., since more time had passed during which care may have been affected).

In addition, linear regression analyses were used to assess whether participants who reported instrumental support exhibited significantly lower depression scores. These analyses were run with the full sample and a second time excluding participants who completed the study survey within the first two weeks of giving birth (*N* = 51 participants), to account for the possibility that the EPDS score was capturing “baby blues” (i.e., short-lasting depressive-like symptoms that may immediately follow delivery) instead of PPD [37]. However, the results did not qualitatively differ between the two models. The full sample was therefore retained to enhance statistical power. All analyses adjusted for maternal age, education, household income, race/ethnicity, postpartum duration, and time between pandemic onset.

## Results

### Sample characteristics and descriptive statistics

Sample descriptive statistics are presented in Table 1. Mean participant age was 32 years old. Most respondents were white (90% of the sample), educated (85% had at least a bachelor’s degree) and had higher income (61% reported an annual household income of $100,000 or more). The majority of participants reported that they were receiving less help and support with household work and/or newborn care (60%) that they attributed to the COVID-19 pandemic; likewise, among the subset of respondents who had other children, many reported their childcare access had been disrupted (57%). Finally, participants varied in depression scores as measured by the EPDS scale, ranging from the minimum score of 0 to 24; the mean EPDS score was 7 and approximately 11% of the sample displayed clinically significant EPDS scores indicative of probable major depression (using a conservative cutoff of > 13) [38].

**Table 1:** Descriptive statistics of model variables

Variable	Mean (SD; range)
Age (years)	31.9 (4.0; 18-47)
Edinburgh Postnatal Depression Survey (EPDS) score	6.96 (4.2; 0-24)
Number of days between onset of pandemic and giving birth	118 (49.9; 13-235)
Number of days between giving birth and completing the study survey	30.7 (12.4; 2-89)
	**Frequency (%)**
Clinically significant EPDS scores	
< 13 (clinical depression unlikely)	868 (89.4%)
> 13 (probable clinical depression)	103 (10.6%)
Race/ethnicity:	
White	872 (89.8%)
Hispanic, Latino, or Spanish origin	42 (4.3%)
Black or African American	10 (1.0%)
Asian	25 (2.6%)
American Indian or Alaskan Native	5 (0.5%)
Other	17 (1.8%)
Household income:	
< $49,999	87 (9.0%)
$50,000 - $99,999	292 (30.1%)
$100,000+	592 (61.0%)
Education level:	
Less than a bachelor’s degree	147 (15.1%)
Bachelor’s degree	342 (35.2%)
Degree beyond a bachelor’s degree	482 (49.7%)
Less support in housework/newborn care due to pandemic	
Yes	582 (59.9%)
No	389 (40.1%)
Childcare access during pandemic (among subset of participants with other children, *n* = 395)	
Affected	227 (57.5%)
Unaffected	168 (42.5%)

Sample means (with standard deviation and range) or frequency (percent) of model variables, for 971 participants included in the analyses

### Postpartum instrumental support in relation to date of giving birth

Logistic regression analyses were carried out to determine whether giving birth later in the course of the pandemic was significantly associated with an increased likelihood of reporting sustained support with household work and newborn care or childcare access (Tables 2 and 3). Preliminary regression analyses assessed whether the likelihood of reporting support varied non-linearly with birth date. No significant non-linear associations were observed in the model assessing the relationship between birth timing and support with household work and newborn care; however, a significant cubic trend was evident between timing of birth in the course of the pandemic and childcare access among the subset of women who had other children (*p* = 0.048).

**Table 2:** Logistic regression model assessing the association between timing of birth during the pandemic and the likelihood of reporting sustained help with household tasks and newborn care

Variable	OR (SE, 95% CI)	p*-*value
Intercept	**6.31 (3.87, 1.90-21.0)**	**0.003**
Age (years)	**0.916 (0.018, 0.882-0.951)**	**< 0.001**
Race/ethnicity:		
White	Reference	
Hispanic, Latino, or Spanish origin	0.588 (0.211, 0.291-1.19)	0.140
Black or African American	**5.54 (3.98, 1.36-22.6)**	**0.017**
Asian	1.98 (0.823, 0.873-4.47)	0.102
American Indian or Alaskan Native	0.627 (0.615, 0.092-4.29)	0.635
Other	2.43 (1.23, 0.901-6.57)	0.079
Household income:		
< $49,999	reference	
$50,000 - $99,999	1.51 (0.407, 0.893-2.56)	0.124
$100,000+	1.33 (0.364, 0.778-2.28)	0.297
Education level:		
Less than a bachelor’s degree	reference	
Bachelor’s degree	**0.563 (0.123, 0.367-0.864)**	**0.009**
Degree beyond a bachelor’s degree	**0.585 (0.130, 0.379-0.904)**	**0.016**
Number of days between giving birth and completing the study survey	1.00 (0.006, 0.990-1.01)	0.789
Number of days between the onset of the pandemic and giving birth	**1.01 (0.001, 1.00-1.01)**	**< 0.001**

Odds ratios are provided with standard errors, 95% confidence intervals, and *p*-values for each variable included in the model

**Table 3:** Logistic regression model assessing the association between timing of birth during the pandemic and the likelihood of reporting continued childcare access, from a subset of participants with other children (*n* = 393). Odds ratios are provided with standard errors, 95% confidence intervals, and *p*-values for each variable included in the model

Variable	OR (SE, 95% CI)	p*-*value
Intercept	0.310 (0.958, 0.001-133)	0.705
Age (years)	0.956 (0.032, 0.897-1.02)	0.183
Race/ethnicity:		
White	reference	
Hispanic, Latino, or Spanish origin	0.899 (0.508, 0.297-2.72)	0.851
Black or African American	1.57 (1.54, 0.231-10.8)	0.643
Asian	1.14 (1.18, 0.152-8.63)	0.896
Other	1.46 (0.333, 0.366-1.81)	0.616
Household income:		
< $49,999	reference	
$50,000 - $99,999	1.65 (0.661, 0.758-3.62)	0.206
$100,000+	0.815 (0.333, 0.366-1.81)	0.616
Education level:		
Less than a bachelor’s degree	reference	
Bachelor’s degree	0.588 (0.205, 0.298-1.16)	0.128
Degree beyond a bachelor’s degree	**0.270 (0.100, 0.130-0.560)**	**< 0.001**
Number of days between giving birth and completing the study survey	0.994 (0.010, 0.974-1.01)	0.581
Number of days between the onset of the pandemic and giving birth:		
Time between	1.10 (0.072, 0.963-1.25)	0.164
Time between squared	0.999 (0.001, 0.998-1.00)	0.094
Time between cubed	**1.00 (1.22e-6, 1.00-1.00)**	**0.048**

In the model assessing the association between time of birth and likelihood of reporting continued help around the house and with newborn care, participants who were older (OR = 0.916, 95%CI: 0.882-0.951, *p* < 0.001) and who were more highly educated (reference: less than a bachelor’s degree; bachelor’s degree OR = 0.563, 95%CI: 0.367-0.864, *p* = 0.009; degree beyond a bachelor’s degree OR = 0.585, 95%CI: 0.379-0.904, *p* = 0.016) were significantly less likely to report sustained instrumental support related to household tasks and newborn care during the pandemic. Conversely, compared to white participants, Black or African American participants were more likely to report that the pandemic had not impacted their support with household tasks and newborn care (OR = 5.54, 95%CI: 1.36-22.61, *p* = 0.017). Finally, as hypothesized, participants who gave birth later in the course of the pandemic were more likely to report that the COVID-19 pandemic had not affected help received with household tasks and newborn care (OR = 1.01, 95%CI: 1.00-1.01, *p* < 0.001) (Table 2). Timing of birth during the pandemic was calculated in days to better capture rapidly shifting shelter-in-place recommendations that may have impacted instrumental support access, while also allowing us to more closely examine the non-linear association between birth timing and childcare access (see below). However, the significant association between birth timing and sustained household help documented here was consistent when timing of birth was calculated in weeks (OR = 1.04, 95%CI: 1.02-1.06, *p* < 0.001) or months (OR = 1.17, 95%CI: 1.08-1.27, p < 0.001).

Among the subset of participants with other children, mothers who were very highly educated were more likely to report disrupted childcare access (reference: less than a bachelor’s degree; degree beyond a bachelor’s: OR = 0.270, 95%CI: 0.130-0.560, p < 0.001). Further, the addition of a cubic term was significant (B = 1.00, 95%CI: 1.00-1.00001, p = 0.048), suggesting a non-linear relationship in childcare access across the course of the pandemic (Table 3). Specifically, the fitted cubic trendline suggested that childcare access increased substantially toward the end of August (~day 170 of the pandemic).

**Fig. 1:**
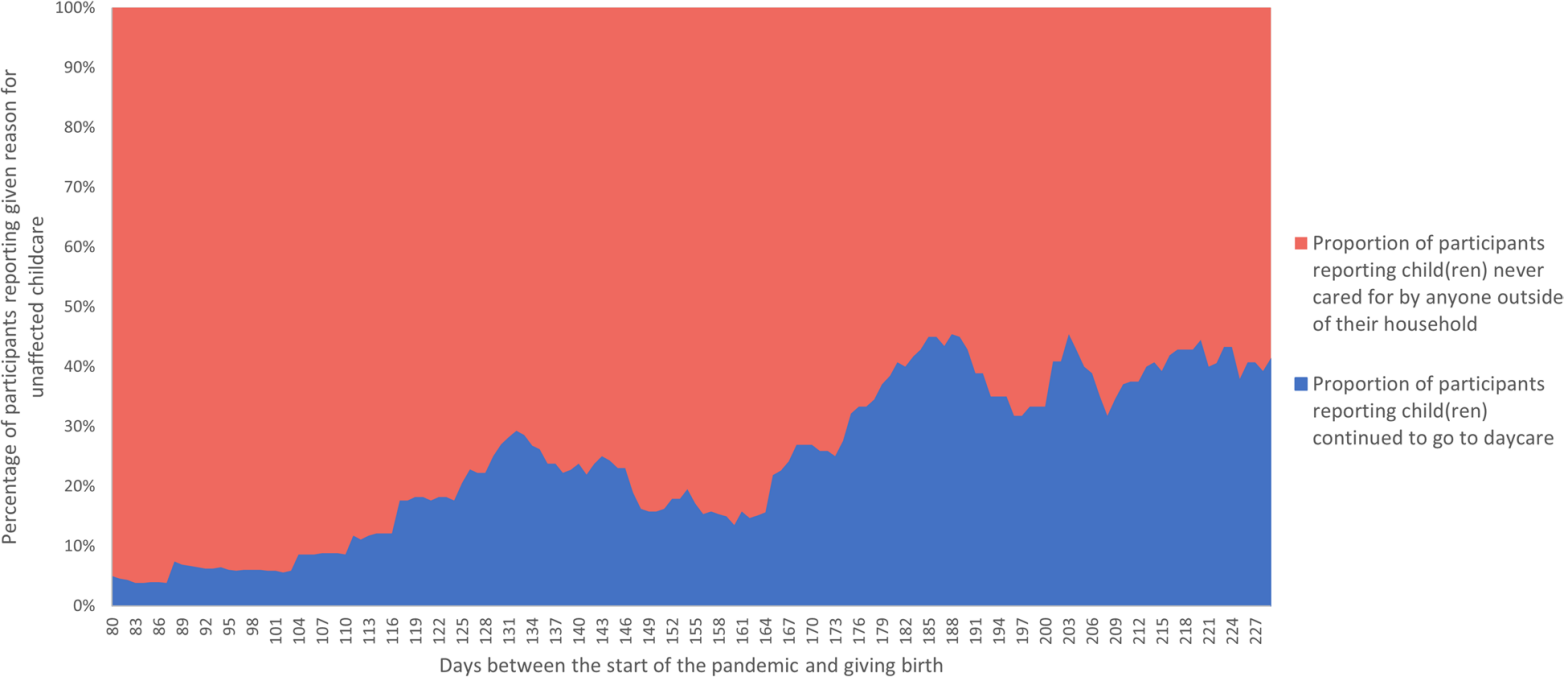
Reason for reporting childcare access was unaffected, plotted as a 30-day moving average

To explore this seemingly paradoxical relationship (i.e., that a greater number of participants reported that the pandemic had not affected their childcare later in the course of the pandemic when more time had passed during which childcare may have been disrupted), moving averages over the previous 30 days were graphed to determine whether response rates for either specific “care unaffected” response were driving this pattern (Fig. 1). Moving (or rolling) averages are derived from successive means over periods of a defined length (e.g., a number of days) and are commonly used to visualize trends over time [39]. A moving average graph of these data suggests that, among individuals reporting sustained access to childcare, the proportion of participants reporting their child(ren) had never been cared for outside of the household generally decreased throughout the pandemic, while the proportion of participants reporting their child(ren) continued to go to daycare simultaneously increased (especially after late August, driving the observed unusual pattern).

The averages reflect the proportion of participants reporting sustained childcare access because child(ren) never cared for by anyone outside of the house (in red) compared to the proportion of participants reporting sustained childcare access because child(ren) continued to go to daycare (in blue) in relation to days between the start of the pandemic and giving birth.

### Postpartum support and maternal depression

Linear regression analyses were carried out to assess whether early postpartum instrumental support was significantly associated with depression score, measured using the EPDS (Tables 4 and 5). Participants who were older (B = − 0.101, 95%CI: − 0.172-(− 0.031), *p* = 0.005), reported a higher household income (reference: < $49,999; $50,000 - $99,999 B = − 1.20, 95%CI: − 2.22-(− 0.180), *p* = 0.021; $100,000+ B = − 1.27, 95%CI: − 2.31-(− 0.236), *p* = 0.016), and were more highly educated (reference: less than a bachelor’s degree; bachelor’s degree B = − 1.06, 95%CI: − 1.90-(− 0.212), *p* = 0.014; degree beyond a bachelor’s degree B = − 1.02, 95%CI: − 1.87-(− 0.159), *p* = 0.020) exhibited significantly lower depression scores. Participants further into their postpartum period (i.e., who exhibited a greater duration of time between giving birth and completing the survey) displayed significantly higher depression scores (B = 0.023, 95%CI: 0.002-0.045, *p* = 0.033). As expected, participants who reported that the pandemic had not disrupted access to help and support with household tasks and newborn care during the pandemic displayed significantly lower depression scores than participants who reported they had received less support due to the pandemic (B = − 1.27, 95%CI: − 1.81-(− 0.731), *p* < 0.001, η^2^ = 0.02) (Table 4).

**Table 4:** Linear regression model assessing the association between reported help with household tasks/newborn care during the COVID-19 pandemic and Edinburgh Postnatal Depression Survey (EPDS) score

Variable	B coefficient (SE, 95% CI)	p*-*value
Intercept	**12.2 (1.19, 9.85-14.5)**	**< 0.001**
Age (years)	**−0.101 (0.036, − 0.172-(− 0.031))**	**0.005**
Race/ethnicity:		
White	reference	
Hispanic, Latino, or Spanish origin	0.981 (0.649, − 0.292-2.26)	0.131
Black or African American	2.25 (1.31, − 0.319-4.82)	0.086
Asian	−0.513 (0.828, − 2.14-1.11)	0.536
American Indian or Alaskan Native	3.15 (1.83, − 0.445-6.74)	0.086
Other	0.662 (0.998, − 1.30-2.62)	0.507
Household income:		
< $49,999	reference	
$50,000 - $99,999	**−1.20 (0.520, − 2.22-(− 0.180))**	**0.021**
$100,000+	**−1.27 (0.528, − 2.31-(− 0.236))**	**0.016**
Education level:		
Less than a bachelor’s degree	reference	
Bachelor’s degree	**−1.06 (0.430, − 1.90-(− 0.212))**	**0.014**
Degree beyond a bachelor’s degree	**−1.02 (0.436, − 1.87-(− 0.159))**	**0.020**
Number of days between giving birth and completing the study survey	**0.023 (0.011, 0.002-0.045)**	**0.033**
Number of days between the onset of the pandemic and giving birth	−0.002 (0.003, − 0.007-0.003)	0.410
Help with household tasks and newborn care affected by the pandemic (yes vs no)	**−1.27 (0.275, − 1.81-(− 0.731))**	**< 0.001**

Beta coefficients are provided with standard errors, 95% confidence intervals, and *p*-values for each variable included in the model

**Table 5:** Linear regression model assessing the association between reported childcare disruptions during the COVID-19 pandemic and Edinburgh Postnatal Depression Survey (EPDS) score, from a subset of participants with other children (*n* = 395). Beta coefficients are provided with standard errors, 95% confidence intervals, and *p*-values for each variable included in the model

Variable	B coefficient (SE, 95% CI)	p*-*value
Intercept	**9.20 (1.99, 5.30-13.1)**	**< 0.001**
Age (years)	−0.085 (0.058, −1.99-0.029)	0.143
Race/ethnicity:		
White	reference	
Hispanic, Latino, or Spanish origin	0.663 (0.999, −1.30-2.63)	0.507
Black or African American	1.06 (1.76, − 2.40-4.51)	0.548
Asian	0.708 (1.96, − 3.15-4.57)	0.718
American Indian or Alaskan Native	4.93 (2.80, − 0.574-10.4)	0.079
Other	−0.665 (1.40, − 3.42-2.09)	0.635
Household income:		
< $49,999	reference	
$50,000 - $99,999	− 0.859 (0.725, − 2.28-0.566)	0.237
$100,000+	−0.937 (0.745, − 2.40-0.527)	0.209
Education level:		
Less than a bachelor’s degree	reference	
Bachelor’s degree	−0.548 (0.627, − 1.78-0.685)	0.383
Degree beyond a bachelor’s degree	−0.799 (0.674, − 2.12-0.526)	0.237
Number of days between giving birth and completing the study survey	**0.056 (0.017, 0.023-0.089)**	**0.001**
Number of days between the onset of the pandemic and giving birth	0.001 (0.005, −0.008-0.010)	0.872
Childcare access (affected vs. unaffected)	**−1.03 (0.421, − 1.86-(− 0.201))**	**0.015**

An additional regression analysis was performed to assess the relationship between depression score and childcare access among the subset of mothers with older children. Participants further into their postpartum period exhibited significantly higher depression scores (B = 0.056, 95%CI: 0.023-0.089, *p* = 0.001). As hypothesized, participants who reported their childcare had not been disrupted exhibited significantly lower depression scores compared to individuals who reported their childcare had been affected by the pandemic (B = − 1.03, 95%CI: − 1.86-(− 0.201), *p* = 0.015, η^2^ = 0.02) (Table 5).

## Discussion

The study findings provide support for both hypotheses. Participants who gave birth later in the course of the pandemic (range April to November 2020) were more likely to report that the pandemic had not affected the support they received with household work and newborn care. This suggests that individuals who gave birth earlier in the pandemic, when lockdowns were more prevalent and general mobility was lower, were more likely to experience disruptions to some aspects of instrumental support. The results also suggest that maintenance of instrumental support during the pandemic may have played an important role in supporting maternal mental health, although it should be noted the effect sizes were rather small. Still, the relationships between the instrumental support measures and PPD score were statistically significant. Specifically, mothers who reported that the pandemic had not negatively impacted their access to childcare or the help they received with housework and newborn care displayed significantly lower depression scores compared to participants who reported pandemic-related disruptions in access to instrumental support. One important strength of the present study is that it focused on protective factors that may improve postpartum wellbeing. Many studies focus on risk factors associated with elevated depression risk [3, 19, 40, 41], including studies examining poor mental health during the COVID-19 pandemic [42, 43]. While this research is important, it is also necessary to frame data analyses and interpretation in a more positive light, including efforts to highlight factors that appear to enhance (rather than reduce) postpartum health.

### Changes in access to instrumental support throughout the course of the pandemic

The results presented here suggest that perceived support related to household work and newborn care has varied throughout the course of the pandemic in the U.S., with participants giving birth later in the pandemic being more likely to report that the pandemic had not affected the social support they received around the house and with newborn care. This pattern may be evident for a couple of reasons. First, participants giving birth later into the pandemic may have benefited from relaxed shelter-in-place restrictions that had by then been instituted in many areas of the country. Mobility data collected using location data stored on Google and Apple devices indicate that mobility in the U.S. was greatly curtailed at the start of the pandemic [28, 29], but that mobility generally increased in the following months as infection spread was reduced or government officials were pressured to ease restrictions as pandemic fatigue increased and citizens were less willing to follow stay-at-home orders [44].

Thus, as shelter-in-place restrictions eased after the first few months of the pandemic in many areas, mothers giving birth during this time may have felt safer receiving support from friends and family around the house during the newborn period. However, this remains to be explicitly tested. Future studies should also test whether individuals in the early postpartum period during the nationwide surge of COVID-19 cases at the end of 2020 and start of 2021 (and associated rise in state restrictions aimed at controlling disease transmission) also report restricted access to childcare and less support around the house, as was documented in the early days of the pandemic when stay-at-home orders were more widespread. In addition to benefiting from relaxed shelter-in-place restrictions, participants who gave birth months into the course of the pandemic in our sample may have also had more time to adjust to the new reality of the pandemic and develop alternative support networks. For example, individuals may have moved in with family members to shelter-in-place together, or they may have formed pandemic “pods” with other families in the area, an idea that has received attention in the media [45, 46]. These new networks may have increased the amount of instrumental support received during the postpartum period. Future work should assess how individuals have shown resilience and shifted their support systems in response to the ongoing pandemic.

However, access to childcare appeared to vary nonlinearly throughout the course of the pandemic, such that higher rates of participants perplexingly reported continual access to childcare later during the pandemic. A graph of moving 30-day averages indicates that a higher percentage of participants with unaffected care reported continued access to daycare later in the pandemic (compared to the percentage reporting that their children had never been cared for by anyone outside the household). It does appear, however, that the rise in respondents indicating that daycare remained unaffected rose noticeably starting at the end of August 2020. It therefore seems likely that this rise could coincide with the start of the school year. School attendance was not explicitly listed as one of the childcare options; it is consequently possible that participants who gave birth later in the course of the pandemic selected the response “my other child(ren) continued to go to daycare” to include children enrolled in school, while respondents earlier in the pandemic were less likely to respond in this manner because older children were out of school due to initial lockdowns or because of summer vacation when American schools are not in session. Additional data collection is needed to explore whether school openings at the start of the academic year were directly related to reports of.

### The importance of instrumental support in supporting maternal mental health

The results of the present study align well with previous work documenting the importance of received support in protecting maternal mental health during the postpartum period. Notably, received support was significantly associated with PPD in this relatively privileged sample (i.e., participants were predominantly white, wealthy, and highly educated); but other work has found social support is especially important in protecting mental health among vulnerable groups (e.g., minoritized communities, individuals with a history of trauma and poor mental health, and those in unstable living conditions or with unreliable healthcare access) [3, 8, 9, 47, 48]. Maternal depression is common during the perinatal period [6], and strong support systems may help buffer against various stressors commonly experienced during this often stressful time. For instance, social support has been shown to enhance maternal self-efficacy, increasing confidence in one’s ability to successfully perform certain behaviors and take on new roles [48]. Moreover, previous evidence suggests that perceptions of social support dampen physiological stress responses by downregulating sympathetic, hypothalamic-pituitary-adrenal (HPA) axis, and inflammatory reactions to stressors [9, 49, 50], thereby dampening the harmful effects of perinatal stress and potentially decreasing the risk of PPD.

However, the ongoing COVID-19 pandemic has dramatically impacted everyday life, leading to widespread perceptions of isolation and reduced social support [51–53]. These changes may disproportionately impact new mothers. Preliminary research has demonstrated that the COVID-19 pandemic has increased reported feelings of loneliness and poor mental health among mothers, as shelter-in-place orders have disrupted daily life and in-person interactions with others [42, 54]. Additionally, the pandemic has also inhibited access to needed support services like childcare, a trend that appears to most strongly affect mothers (compared to fathers or other caregivers), especially working mothers [23, 24]. Research prior to the pandemic indicates that new mothers who report greater levels of work spillover into the home exhibited lower mental health scores and that assistance in newborn care from family members was a consistent predictor of wellbeing [11, 55]. It therefore seems likely that the blurring of work-home boundaries during the pandemic has had a negative impact on maternal mental health, while reduced help around the house during the postpartum period and unreliable access to childcare may compound this issue and increase PPD risk.

Interestingly, timing of giving birth in the course of the pandemic was not significantly related to maternal PPD score in either model. It is possible that this lack of an association is due to the pandemic exerting different effects on mental health as the COVID-19 pandemic persists over time. For instance, mothers may experience an increased risk for depression early in pandemic in response to initial disruptions to daily life and COVID-19-related feelings of panic and uncertainty. Yet, while the initial negative emotions and social disruptions may have partly subsided over time, elevated maternal depression risk may have persisted as individuals instead suffered from pandemic fatigue and/or financial worries [4, 56, 57]. Future research is needed to assess how specific factors contributing to maternal depression risk during the COVID-19 pandemic may vary over time.

### Maternal education level and pandemic-related changes in support

The risk of poor mental health outcomes, including PPD, is not uniformly experienced across all groups [9, 58, 59]. Previous work suggests that socioeconomic status (SES) is a significant predictor of PPD risk, such that individuals of low SES exhibit the greatest risk of PPD [58, 59]. Socioeconomic status is a summary measure of individual social and economic position in relation to others and is shaped by many factors, including income and education level. Specifically, higher education and income levels are associated with increased SES, and also with lower PPD risk [58, 59]. Higher SES may help buffer against depression through reducing the stressors mothers face on a daily basis, while also facilitating access to support networks (e.g., hired help for housework, in-home childcare providers, mother-infant activity groups such as baby yoga, etc.) [60, 61]. However, the benefits associated with higher SES with regards to mental health may be diminished during the COVID-19 pandemic.

For example, income level was not associated with increased likelihood of sustained help with household work and newborn care or continued access to childcare. Conversely, education level appeared to be more consistently related with disrupted instrumental support, but not in the expected direction. More highly educated participants were more likely to report that they were receiving less help with housework and newborn care due to the pandemic, and were also more likely to indicate that their access to childcare had been disrupted. One possible explanation for this surprising pattern is that the pandemic has more strongly impacted nonparental instrumental support utilized by well educated, high SES individuals (e.g., hired help and nonparental childcare such as nannies or daycares) [62, 63]. For instance, well educated, high SES parents are likely better able to afford the high costs of non-parental childcare in the U.S., allowing mothers to continue working [64, 65]. It is also possible that more highly educated individuals rely on these services in part because they are more likely to live farther away from familial support systems [66, 67].

Evidence suggests that a positive trend exists between education level and relocation due to work-related reasons (as opposed to family-related or housing-related reasons), with highly educated individuals tending to relocate to areas with more employment opportunities [66, 67]. In other words, mothers with higher education levels may relocate far from family for work-related reasons more often than individuals with less formal education. While additional work is needed to test this hypothesis, preliminary analyses using the CARE study database has documented a positive association between education level and the likelihood of participants reporting that their loved ones were unable to meet their infant due to the pandemic (unpublished data), suggesting that highly educated participants may live farther from family members who were unable to safely travel during the pandemic to provide support during the postpartum period. As has been documented elsewhere [68, 69], it is also possible that more high educated participants in this sample were more likely to comply with recommended preventive measures; thereby decreasing the likelihood of allowing loved ones to visit or using childcare services outside of the home. Future studies should explore how available instrumental support during the pandemic may vary by SES measures, such as education level.

In addition to SES measures, additional work using more diverse samples is required to examine how race/ethnicity may be associated with employment and instrumental support access during the pandemic. People of color exhibit greater exposure to psychosocial and economic stressors, increasing their PPD risk [9, 59]. Communities of color have also been disproportionately affected by the COVID-19 pandemic, with higher morbidity and mortality rates [70–72]. Additionally, individuals of color are overrepresented among low-wage essential workers; positions which require parents to continue working outside the home during the pandemic, a challenging prospect when childcare services remain closed [73, 74]. It is therefore critical to assess how the ongoing pandemic may affect PPD risk in minority populations and determine how individuals may draw on existing or novel support networks to buffer against pandemic-related stressors. Previous work indicates that enhanced social support may decrease the risk of PPD in people of color [9], suggesting that fostering strong support networks may represent an important non-pharmaceutical strategy to support mental health during the postpartum period across diverse communities. Future work should explore examples of resilience and social support during the COVID-19 pandemic.

### Healthcare and policy implications

Research prior to the COVID-19 pandemic suggests that PPD goes undiagnosed in one of every five to eight postpartum individuals, translating to more than a half a million individuals going undiagnosed each year [10, 75]. This pattern may be due in part to a lack of PPD screening. Results of a multiple-site study (31 sites) in the U.S. revealed that one in eight individuals with a live birth reported not being asked about depression during a postpartum visit [75]. More consistent screening protocols are therefore needed, especially during the COVID-19 pandemic as PPD levels rise, to correctly identify PPD cases in order to initiate appropriate care. In addition to screening more frequently, providers and policymakers should consider which factors may either increase or decrease PPD risk, both during and after the COVID-19 pandemic.

For example, the results presented here cumulatively suggest that medical care providers should consider sustained postpartum instrumental support as a strategy to support maternal mental health during the pandemic. Interventions that enhance support may consequently offer an efficient, non-pharmaceutical technique to protect maternal mental health and reduce depression risk [41], especially if combined with other interventions. Thus, policies designed to reduce disease transmission and allow individuals to safely interact with others -- such as investing in widespread and regular COVID-19 testing -- may reduce disruptions to instrumental support received during the postpartum period and reduce the risk of PPD (i.e., by allowing childcare services to remain safely open and help mothers feel comfortable allowing individuals with negative tests to visit and help with household work and newborn care). Yet, any novel PPD interventions during the COVID-19 pandemic should also consider how the pandemic may impact individuals differently, with implications for disrupted support networks and subsequent poor mental health outcomes.

### Limitations

It should be noted that despite the strengths of these analyses (e.g., large sample size and participants from across the U.S.), several important study limitations exist. First, as mentioned above, the study survey did not explicitly ask about in-person school attendance as a form of childcare access, and it is unclear how participants may have reported this type of childcare. Likewise, although we included several relevant confounders in the statistical models, PPD is a complex condition with many possible confounders (e.g., previously experienced trauma and poor health, additional markers of socioeconomic status such as neighborhood disadvantage, etc.). It is consequently likely these models failed to account for all relevant factors that influence PPD risk because these data were not collected in the study survey. Future studies should expand upon these analyses and include additional possible confounders during statistical analysis. In addition, this study is cross-sectional. It is therefore not possible to definitively determine whether the significant relationship observed between reported social support and maternal depression score is due to instrumental support protecting against depression or to maternal depression altering perceptions of received instrumental support. Longitudinal data collection is needed to establish causal relationships. In addition, due to the use of convenience sampling, these data are not representative of the U.S. population as a whole; white, educated, wealthy individuals are overrepresented in the present sample compared to the U.S. birthing population [76]. Additional work is needed to determine whether the associations observed here are also evident across a more representative, diverse sample of the U.S. population.

## Conclusions

The COVID-19 pandemic has significantly disrupted instrumental support systems, including for individuals in the postpartum period. Postpartum recovery is bolstered by assistance from others, via help with household chores, newborn care, and watching other children. However, typical systems of support -- including family, friends, and paid help -- may have been impacted by the pandemic due to social distancing mandates. Our findings indicate that the likelihood of reporting uninterrupted help with housework, newborn care, and childcare for older children in the early postpartum period has changed over time. Respondents who gave birth later in the course of the pandemic were more likely to indicate that the pandemic had not affected the help they received with household work and newborn care, suggesting that participants who gave birth earlier in the pandemic were more likely to experience disruptions to these aspects of social support (potentially due to the more restrictive shelter-in-place orders evident at the start of the pandemic). Access to childcare also varied over time, although this relationship was nonlinear and suggested that a higher proportion of participants reported continued access to childcare beginning in late August, perhaps coinciding with the start of the school year and students returning to in-person classroom instruction.

Our results also suggest that social support systems known to protect against PPD may be especially important in supporting mental health during the pandemic, such that respondents who reported that the pandemic had not disrupted instrumental support with household work and newborn care exhibited lower depression scores. Likewise, among a subset of participants with other children, continued access to childcare during the pandemic was associated with lower depression scores. Instrumental support during the postpartum period therefore represents a potential tool to protect against PPD, both during and after the COVID-19 pandemic.

## Data Availability

The datasets used and analyzed during the current study are available from the corresponding author upon reasonable request.

## Abbreviations

COVID-19: Coronavirus Disease 2019
EPDS: Edinburgh postnatal depression survey
PPD: Postpartum depression
